# The Association between Bone Quality and Atherosclerosis: Results from Two Large Population-Based Studies

**DOI:** 10.1155/2017/3946569

**Published:** 2017-08-09

**Authors:** V. Lange, M. Dörr, U. Schminke, H. Völzke, M. Nauck, H. Wallaschofski, A. Hannemann

**Affiliations:** ^1^Institute of Clinical Chemistry and Laboratory Medicine, University Medicine Greifswald, Greifswald, Germany; ^2^Department for Internal Medicine B, University Medicine Greifswald, Greifswald, Germany; ^3^German Centre for Cardiovascular Research, Partner Site Greifswald, Greifswald, Germany; ^4^Department for Neurology, University Medicine Greifswald, Greifswald, Germany; ^5^Institute for Community Medicine, University Medicine Greifswald, Greifswald, Germany

## Abstract

**Objective:**

It is highly debated whether associations between osteoporosis and atherosclerosis are independent of cardiovascular risk factors. We aimed to explore the associations between quantitative ultrasound (QUS) parameters at the heel with the carotid artery intima-media thickness (IMT), the presence of carotid artery plaques, and the ankle-brachial index (ABI).

**Methods:**

The study population comprised 5680 men and women aged 20–93 years from two population-based cohort studies: Study of Health in Pomerania (SHIP) and SHIP-Trend. QUS measurements were performed at the heel. The extracranial carotid arteries were examined with B-mode ultrasonography. ABI was measured in a subgroup of 3853 participants. Analyses of variance and linear and logistic regression models were calculated and adjusted for major cardiovascular risk factors.

**Results:**

Men but not women had significantly increased odds for carotid artery plaques with decreasing QUS parameters independent of diabetes mellitus, dyslipidemia, and hypertension. Beyond this, the QUS parameters were not significantly associated with IMT or ABI in fully adjusted models.

**Conclusions:**

Our data argue against an independent role of bone metabolism in atherosclerotic changes in women. Yet, in men, associations with advanced atherosclerosis, exist. Thus, men presenting with clinical signs of osteoporosis may be at increased risk for atherosclerotic disease.

## 1. Introduction

Osteoporosis and atherosclerosis substantially impact the elderly, leading to increased morbidity and mortality [1, 2]. Osteoporosis, on the one side, is a chronic disease characterized by low bone mass, microarchitectural deterioration of bone tissue, and an increased fracture risk [1]. Based on these diagnostic criteria, 27.6 million women and men in the European Union are affected by osteoporosis [1]. Atherosclerosis, on the other side, is characterized by loss of elasticity of the artery walls, wall thickening, and plaque formation [2]. Cardiovascular disease due to atherosclerosis represents a major economic burden on the European health care system, with estimated annual costs of 192 billion euros [3]. Moreover, cardiovascular disease is the leading cause of death worldwide, with more than 17.5 million deaths in 2012 [4].

Osteoporosis and atherosclerosis share common risk factors like aging, dyslipidemia, oxidative stress, inflammation, hypertension, and diabetes [2, 5]. Furthermore, they share molecular pathways involving, for example, bone and vascular mineralization or inflammatory processes [6]. Despite these common risk factors, epidemiologic studies suggested an independent association between low bone mass and atherosclerosis [5, 7]. Thus, a population-based study including [5] 2726 postmenopausal women and 2543 men from Norway reported associations between low bone mineral density (BMD) and echogenic calcified atherosclerotic plaques. Further, lower volumetric trabecular lumbar BMD was associated with advanced carotid plaque in 1833 postmenopausal women and men from a subsample of the Multi-Ethnic Study of Atherosclerosis [7]. In that study [7], also, associations of lower volumetric trabecular lumbar BMD with lower ankle-brachial index (ABI) and increased internal carotid artery intima-media thickness (IMT) were found in men but not in women. A male-specific inverse association between femoral neck BMD and the 10-year risk for coronary heart disease was also described in a study including 5415 men and 7409 women from the general population in Korea [8]. On the other side, associations between BMD and pulse wave velocity [9] as well as between BMD loss and coronary artery calcification [10] were described for women only. The previous study results [5, 7–10] appear even more controversial when considering that competing studies reported no associations between a low BMD and carotid IMT as well as pulse wave velocity and ABI [11] or that associations between peripheral artery disease and bone loss turned nonsignificant after age adjustment [12].

Taken together, it is still uncertain whether low bone quality is associated with atherosclerotic changes independent of major cardiovascular risk factors like diabetes mellitus, dyslipidemia, or hypertension or whether sex-specific differences exist. Besides, there are only few data in the association between bone quality at the heel, measured by quantitative ultrasound (QUS), and atherosclerosis [13, 14]. We therefore aimed to explore the associations between QUS-based parameters, with carotid artery IMT, the presence of carotid artery plaques, and the ABI in the general population.

## 2. Subjects and Methods

### 2.1. Study Populations

The present analysis is based on data from two population-based cohort studies in northeast Germany: the second follow-up of the Study of Health in Pomerania (SHIP-2) and SHIP-Trend. Details on the study design, protocols, and sampling methods have been reported elsewhere [15, 16]. In short, the baseline examinations in the SHIP cohort were performed between 1997 and 2001 with a total of 4308 men and women aged 20–81 years. The second follow-up (SHIP-2) was performed between 2008 and 2012 with 2333 subjects aged 30–93 years being re-examined. The baseline examinations in the SHIP-Trend cohort were performed in parallel with SHIP-2 between 2008 and 2012 with a total of 4420 adult men and women aged 20–84 years. All investigations were carried out in accordance with the Declaration of Helsinki, including written informed consent of all participants. The study methods were approved by an institutional review board (ethics committee at the University of Greifswald).

Data from SHIP-2 and SHIP-Trend were pooled for the present analyses. From the resulting population of *N* = 6753, all subjects with missing data in QUS, IMT, plaque, or confounder variables were excluded as well as subjects with extremely high values in the QUS variables, pregnant women, subjects with estimated glomerular filtration rate (eGFR) below 30 ml/min/1.73m^2^ or missing eGFR, and those who reported with intake of bisphosphonates, selective estrogen receptor modulators, parathyroid hormone, steroids, or strontium ranelate (for details, see Supplemental Figure 1 available online at https://doi.org/10.1155/2017/3946569). The resulting study population comprised 5680 subjects. Among these subjects, two-thirds (67.8%) participated in the ABI examination, resulting in a subsample of 3853 subjects.

### 2.2. Interview and Physical Examination

All SHIP-2 and SHIP-Trend participants were offered a large range of standardized medical examinations, biomaterial sampling, and an extensive computer-aided personal interview. During the personal interview, information on sociodemographic characteristics, lifestyle, and medical histories were collected. All participants were asked to bring their medications taken seven days prior to the time of examination. Medication data were obtained online using the IDOM program (online drug-database leaded medication assessment) and classified using the Anatomical-Therapeutic-Chemical (ATC) classification system. Intake of bisphosphonates was defined as ATC M05BA-BB, selective estrogen receptor modulators as ATC G03XC, PTH preparations as ATC H05AA, strontium ranelate as ATC M05BX03, glucocorticoids for systemic use as ATC H02AB and H02BX, vitamin D preparations as ATC A11CC, and cardioprotective medication as ATC C. Participants were defined as physically inactive if they reported less than one hour of regular physical activity per week during summer and winter. Risky alcohol consumption was defined as alcohol intake at or above 30 g/day in men and 20 g/day in women. All women older than 60 years of age and women between 40 and 60 years of age without self-reported menstrual cycling were classified as postmenopausal. Years since menopause were calculated as the difference between current age and age at last menstruation. Standardized measurements of body height and weight were performed with calibrated scales. Body mass index (BMI) was calculated as weight (kg)/height^2^ (m^2^). Systolic and diastolic blood pressures were measured three times on the right arm of seated subjects, using a digital blood pressure monitor (HEM-705CP, Omron Corporation, Tokyo, Japan). The mean of the second and third measurements was used for statistical analyses. Hypertension was defined as systolic blood pressure ≥ 140 mmHg or diastolic blood pressure ≥ 90 mmHg or self-reported intake of antihypertensive medication. Diabetes mellitus was defined when a respective physician's diagnosis or intake of antidiabetic medication (ATC A10) was reported, when HbA1c was ≥6.5% or serum glucose concentrations were ≥11.1 mmol/l. Dyslipidemia was defined as total cholesterol concentration ≥ 6.2 mmol/l or LDL-cholesterol concentration ≥ 4.1 mmol/l or HDL-cholesterol concentration < 1.04 mmol/l or triglycerides ≥ 1.7 mmol/l or intake of lipid-modifying agents (ATC C10).

### 2.3. QUS

QUS at the heel was performed using the Achilles InSight device (GE Medical Systems Ultrasound, GE Healthcare, Chalfont St. Giles, U.K.), a water-based bone ultrasonometer, as reported previously [17]. Two devices without systematic differences were used during the course of the study. The measurements were performed successively on both feet of the seated participants by trained and certified examiners. Alcohol was used as a coupling agent. The system measures the frequency-dependent attenuation of the sound waves (broadband ultrasound attenuation (BUA)) and the speed of sound waves (SOS) as they pass through the heel (os calcis). BUA and SOS were combined to form the stiffness index according to the following formula: stiffness index = (0.67 × BUA) + (0.28 × SOS) − 420. The system automatically compares individual stiffness index results to values obtained in a healthy young reference population. Indices below the reference mean minus 2.5 standard deviations were taken to indicate a high osteoporotic fracture risk, indices above the reference mean minus 2.5 standard deviations but below the reference mean minus 1 standard deviations were taken to indicate a medium osteoporotic fracture risk, and indices above the reference mean minus 1 standard deviations were taken to indicate a low osteoporotic fracture risk [17].

### 2.4. IMT and Plaques

Certified medical assistants examined the extracranial carotid arteries with B-mode ultrasonography (vivid-i, GE Medical Systems, Waukesha, WI, USA) using a broad-bandwidth linear array transducer with an operating frequency of 13 MHz. Longitudinal scans of the distal straight portion of the far wall of the common carotid artery (CCA) of both sides were recorded. CCA-IMT was assessed on-screen using a semiautomated edge tracking software, which measures the distance between the lumen-intima and media-adventitia interfaces at an arterial segment of 1 cm in length located directly proximal to the widening of the artery at the bifurcation. The “mean CCA-IMT,” which was used for statistical analyses, was calculated as the average of the mean values of 250 measurement points of each side. The carotid arteries were further evaluated in longitudinal and cross-sectional scans for the presence of atherosclerotic plaques. Each arterial segment (i.e., the left or right common carotid artery, internal carotid artery, external carotid artery, and the carotid bifurcation) was categorized into either affected by plaque or plaque-free. If at least one arterial segment was classified as being affected by plaque, “carotid plaques” were defined as being present. Further, the number of arterial sites affected by plaque ranging between zero and eight was recorded.

### 2.5. ABI

The Doppler method was used to determine systolic blood pressure in both arms (brachial artery) and both ankles (anterior and posterior tibial arteries) for the calculation of the ABI. After at least ten minutes of rest in the supine position, the measurements were started. The measurements were performed with the “Dopplex D900” (Huntleigh Healthcare Ltd., Cardiff, U.K.) and a blood pressure cuff (Welch Allyn, Skaneateles Falls, USA). The calculation of the ABI followed the guidelines of the American Heart Association [18]. The higher of the anterior and posterior tibial artery systolic blood pressure of each leg was divided by the higher of the right or left brachial artery systolic blood pressure. The lower of the right or left leg ABIs was used for statistical analyses.

### 2.6. Laboratory Measurements

Blood samples were taken between 7 a.m. and 1 p.m. from participants in the supine position. Creatinine, glucose, total cholesterol, triglyceride, HDL-cholesterol, and LDL-cholesterol serum concentrations were measured on the Dimension Vista (Siemens Healthcare Diagnostics, Eschborn, Germany). Hba1c concentrations were measured with high performance liquid chromatography on a DIAMAT analyzer (Bio-Rad Laboratories, Munich, Germany). Serum 25-hydroxy vitamin D (25OHD) concentrations were measured in a subgroup of 3540 (62.3%) SHIP-Trend participants with the IDS-iSYS 25-hydroxy vitamin D assay on the IDS-iSYS Multidiscipline Automated Analyser (Immunodiagnostic Systems Limited, Frankfurt am Main, Germany). The eGFR was calculated according to the 4-variable modification of diet in renal disease equation [19].

### 2.7. Statistical Analyses

Due to previously reported marked differences between men and women in the association between bone metabolism and atherosclerotic changes, all analyses were stratified by sex. Continuous data are expressed as median (1st–3rd quartiles) and nominal data as percentage. The Kruskal-Wallis test or the *χ*^2^ test was used for group comparisons. A value of *p* < 0.05 was considered statistically significant.

Analyses of variance (ANOVA) and linear and logistic regression analyses were performed to assess the associations of the QUS-based parameters (exposures: BUA, SOS, stiffness index, and risk for osteoporotic fractures) with the cardiovascular parameters (outcomes: IMT, carotid plaque, the number of arterial segments, and ABI). In all analyses, a one standard deviation decrease in the continuous exposure variables (BUA 13.7 and 14.7 dB/MHz; SOS 37.5 and 33.5 m/s; and stiffness index 18.1 and 17.5 in men and women, resp.) was modelled. In models with the categorical exposure variable, osteoporotic fracture risk, a low osteoporotic fracture risk was used as a reference category. The outcomes IMT and ABI entered the regression models as continuous variables, while carotid plaque was dichotomized (present/not present). Finally, the number of arterial segments affected by plaque was used as a continuous variable. It was transformed (log (number of segments + 1)) before being entered in the ANOVA or linear regression models. We report adjusted means with 95% confidence intervals from the ANOVA, *β*-coefficients with standard errors and *p* values from the linear regression models and odds ratios with 95% confidence intervals from the logistic regression models. Results from unadjusted and fully adjusted models, including age, BMI, smoking status, physical inactivity, risky alcohol consumption, diabetes mellitus, dyslipidemia, hypertension, and, in women, intake of estrogens (oral contraceptives or hormone replacement therapy) and years since menopause, are presented.

Among the SHIP-2 and SHIP-Trend participants, a subpopulation underwent the ABI examination. To account for the possible selection bias according to nonparticipation, inverse probability weights using sex, age, smoking, education, equivalence household income, blood pressure, antihypertensive medication, diabetes mellitus, HbA1c, lipids, and BMI as explanatory variables were applied [20]. All statistical analyses were performed with SAS 9.4 (SAS Institute Inc., Cary, North Carolina, USA).

## 3. Results

The majority of men (68.2%) and women (61.2%) in our study population had a low QUS-based osteoporotic fracture risk. On the other side, 4.4% of men and 6.5% of women had a high and 27.4% and 32.3% a medium QUS-based osteoporotic fracture risk, respectively. The presence of cardiovascular risk factors differed between the three fracture risk groups. Men with a high fracture risk were older, had lower BMI, were more often smokers or physically inactive, and were more often risky alcohol consumers than men with a low or medium fracture risk. Women with a high risk were also older than those with a low or medium risk, had more often diabetes, dyslipidemia, and hypertension but did not differ with respect to BMI, physical inactivity, and risky alcohol consumption. Besides, the CCA-IMT, the presence of plaques, and the number of arterial segments affected by plaque significantly increased over the three risk groups in men and women (Table 1).

In unadjusted linear regression analyses, inverse associations of BUA, SOS, stiffness index, or the QUS-based osteoporotic fracture risk with the CCA-IMT were found. In men, for example, a decrease in the stiffness index by 18.1 points was associated with an increase in CCA-IMT of 0.016 cm. After adjustment for age (data not shown) and also in fully adjusted models, however, the associations were not confirmed (Table 2).

**Figure 1:**
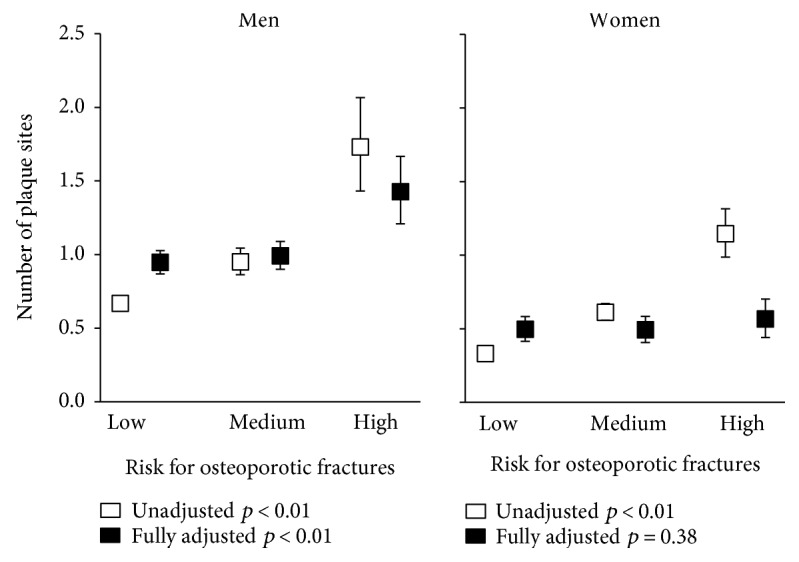
Adjusted mean number of plaque sites according to QUS-based osteoporotic fracture risk by sex. ANOVA was adjusted for age, body mass index, smoking status, physical inactivity, risky alcohol consumption, diabetes mellitus, dyslipidemia, hypertension, and, in women, additionally intake of estrogens (oral contraceptives or hormone replacement therapy) and years since menopause. The number of arterial segments affected by plaque was transformed (log (number of plaque sites + 1)) before being entered in the model and back-transformed for display in the figure.

Regarding plaques, unadjusted logistic regression analyses demonstrated significantly increased odds for the presence of plaques with decreasing QUS-based parameters in both sexes. In fully adjusted models, these associations were confirmed in men but not in women (Table 3). Men with a high fracture risk had significantly increased odds for plaque occurrence compared to men with a low risk. In men, also, the number of arterial segments affected by plaque significantly increased over the risk categories (Figure 1) in unadjusted as well as in the fully adjusted ANOVA. In women, similar findings were made in unadjusted analyses, whereas in fully adjusted models, the results disappeared.

In the subpopulation with ABI measurement, the proportions of men and women with high, medium, or low fracture risk were comparable to the proportions in the whole population (men and women high risk: 69.2% and 62.1%; medium risk: 26.3% and 32.0%; and low risk: 4.5% and 5.9%; for more details, see Supplemental Table 1). The ABI was similar between the three risk groups [median (1st–3rd quartiles) for men and women with a high risk: 1.12 (1.07–1.19) and 1.11 (1.07–1.18), medium risk: 1.13 (1.07–1.19) and 1.13 (1.08–1.19), and low risk: 1.12 (1.04–1.21) and 1.13 (1.08–1.18), resp.]. Fully adjusted, sex-specific linear regression models (Supplemental Table 2) revealed no significant association between the QUS parameters and the ABI in men or women.

## 4. Discussion

In the present study, we demonstrated associations between decreased bone quality, defined by heel QUS parameters, and the presence of atherosclerotic carotid artery plaque in men. These associations were independent of major cardiovascular risk factors, including dyslipidemia, diabetes, and hypertension. In contrast, our data do not provide evidence for relevant independent associations of heel bone quality with CCA-IMT or ABI.

Traditionally, osteoporosis and atherosclerosis have been regarded as independent processes sharing common risk factors, for example, aging [21]. Recent evidence from cell culture as well as epidemiological studies, however, points to an age-independent association between osteoporosis and atherosclerosis [6, 22]. Defects in bone mineralization and arterial calcification were attributed of having a similar pathogenesis [6], and associations between bone quality and atherosclerotic changes independent of cardiovascular risk factors have been proposed.

Following this hypothesis, the previous studies [23, 24] demonstrated associations between decreased BMD and increased IMT in elderly individuals. For example, the San Antonio Family Osteoporosis Study demonstrated that decreased BMD at various sites is correlated with carotid artery IMT in older women and men [23]. Among the SHIP-2 and SHIP-Trend participants, highly significant inverse associations between the QUS-based parameters and the carotid artery IMT were observed in unadjusted models but turned nonsignificant after adjustment for cardiovascular risk factors. Comparable observations were made by Frost et al. [25], who found associations between spine BMD and IMT, but the relationship was not significant after adjustment for age, mean arterial pressure, and triglycerides.

While our data thus argues against independent associations between bone metabolism and IMT, it provides evidence for a male-specific relation with carotid artery plaques. CCA-IMT and plaques are biologically and genetically distinct markers of atherosclerosis. They differ with respect to specific pattern of risk factors, their pathogenesis, and their ability to predict cardiovascular and cerebrovascular events [26]. Numerous factors including inflammation, protein metabolism, and oxidative stress were proposed to promote development of disease in both osteoporotic and atherosclerotic changes [7]. These factors are likely involved in the pathogenesis of focal atherosclerotic plaques but are less important for an arterial wall thickening of the CCA, which may explain the strong association between QUS-based bone quality and plaque occurrence and the nonsignificant association between bone quality and IMT. Corresponding observations of associations between bone quality and the presence of plaques were reported from various studies [5, 25, 27, 28]. For example, the Tromsø study [5] showed that BMD was associated with calcified echogenic plaque independent of potential confounders, mediators, and shared risk factors in men and women [5]. Also, Hyder et al. investigated in 904 postmenopausal women and 929 men an independent association between echogenically measured carotid plaque and lower volumetric trabecular lumbar BMD [7]. While our results were restricted to men, other studies reported associations between carotid plaques and BMD only in women [10, 27] or were restricted to women [25, 29, 30]. Nevertheless, also, male-specific associations between BMD and plaques [7, 8, 31, 32] were previously reported.

The sex differences in the examined associations may be explained by differences in bone metabolism between men and women. Men have a higher peak of bone mass than women [33], and osteoporosis emerges later in life and is more often due to secondary causes [34]. At the same time, osteoporosis is strongly associated with comorbidities and frailty in men than in women [35] and men have a higher fracture-related mortality than women [36]. Thus, men presenting with clinical signs of osteoporosis may be generally more frail and present with a larger number of comorbidities than men without such signs. This probably explains the increased risk of cardiovascular pathologies with decreasing bone quality, independent of age, and further cardiovascular risk factors. In these men, heightened awareness regarding atherosclerotic changes may be of clinical importance.

In previous studies, bone quality was rarely assessed by QUS, instead dual-X-ray-absorptiometry (DXA) was performed. DXA provides information on BMD and is the recommended measurement in the diagnosis and monitoring of osteoporosis [37, 38]. However, also, QUS provides reliable results to predict fracture risk [39, 40] and has the advantage of being radiation-free and easy to handle. To our knowledge, there are only few studies using QUS to investigate the association between bone quality and atherosclerosis [13, 41–43]. These studies reported conflicting results, but predominantly suggest an association between QUS and atherosclerotic changes, which is in line with our results.

Next to IMT and plaques, we assessed the relation between bone stiffness and ABI, as a marker of vascular calcification and increased vessel stiffness [44]. It is known that decreased blood flow to the lower limbs caused by PAD leads to compromising the bone quality [45]. In line with this, the Rotterdam Study [46], including 5268 individuals, reported a significantly increased risk for PAD in women with a low femoral neck BMD even after adjusting for age. Our study failed to show an association between bone stiffness and ABI. Yet, PAD as defined by ABI ≤ 0.9 was rare in our study population; only 1.53% of participants entailed these values, and early stages of PAD may not impair bone health [12]. This may have prevented us from detecting a respective association. Moreover, our results are in line with the majority of previous studies that report no [43, 47] or only weak [12, 48] associations between BMD and arterial stiffness.

Our study has several strengths and limitations. Strengths result from the large sample including men and women over a large age range (20–93 years). Further, all study participants underwent intensive medical examinations with highly standardized procedures, assuring high data quality. Moreover, we adjusted our models for interfering covariates to assess the impact of comorbidities.

Besides these strengths, our study has its limitations. First, the cross-sectional design does not allow assessing causality between the measures. Second, cardioprotective drugs were taken by a large proportion (40.7%) of our study population. The intake of such medication reduces the cardiovascular risk and may lead to an underestimation of the effect of the examined associations. Third, BMD measurements were not available; thus, our study is not directly comparable to other studies using BMD. However, the QUS-based results provide complementary evidence and, in a population-based research setting, offer the advantage of being simpler, less expensive, and free of ionizing radiation. Fourth, ABI measurements were only available in a subsample. To rule out that nonparticipation resulted in a selection bias, we weighted the respective data based on social-demographic and health-related variables. Fifth, 25OHD concentrations were unavailable in nearly 40% of the study population, and vitamin D intake was reported by only 29 subjects. Therefore, we refrained from including that information in the analyses. Sixth, our study was performed exclusively in Caucasian European subjects; thus, our results may not be directly transferrable to other regions or ethnicities.

In conclusion, our data argue against an independent role of bone metabolism in atherosclerotic changes in women. Yet, in men, associations with atherosclerotic changes, especially formation of plaques, seem present. Thus, men presenting with clinical signs of osteoporosis may be at increased risk for atherosclerotic disease. Further studies are needed to understand the relation between calcified plaque and decreased bone quality.

## Supplementary Material

Supplemental Figure 1. Selection of the study populations. Supplemental Table 1. Characteristics of the ABI study population. Supplemental Table 2. Associations between a decrease in QUS-based parameters and ABI.

## Figures and Tables

**Table 1:** Characteristics of the IMT study population.

Characteristics	Risk for osteoporotic fractures—men				Risk for osteoporotic fractures—women			
	Low (*n* = 1887)	Medium (*n* = 759)	High (*n* = 122)	*p*	Low (*n* = 1783)	Medium (*n* = 940)	High (*n* = 189)	*p*
Age, years	52 (41–64)	56 (45–68)	63 (52–71)	<0.01	47 (38–58)	58 (47–67)	67 (59–75)	<0.01
BMI, kg/m^2^	28.2 (25.8–31.1)	27.8 (25.1–30.5)	27.6 (24.5–30.7)	<0.01	26.5 (23.3–30.8)	26.4 (23.3–30.3)	26.7 (23.7–29.5)	0.59
Current smoker, %	24.1	31.6	38.5	<0.01	26.1	21.4	17.5	<0.01
Physically inactive, %	48.6	57.8	63.9	<0.01	51.1	48.2	50.3	0.35
Risky alcohol consumption, %	13.3	16.1	20.5	0.02	3.0	2.6	2.7	0.81
25OHD, ng/ml^∗^	23.4 (17.9–29.6)	22.7 (16.9–28.2)	20.4 (16.2–28.9)	0.08	22.4 (16.4–29.8)	22.8 (16.8–29.2)	20.5 (15.0–28.1)	0.25
Diabetes mellitus, %	12.8	16.5	14.8	0.04	8.8	11.4	16.4	<0.01
Dyslipidemia, %	67.4	71.4	71.3	0.10	48.6	58.5	67.7	<0.01
Hypertension, %	71.1	74.3	75.4	0.18	43.2	55.5	72.0	<0.01
BUA, dB/MHz	123 (116–131)	105 (101–109)	94 (89–97)	<0.01	116 (109–125)	99 (94–103)	86 (82–90)	<0.01
SOS, m/s	1576 (1560–1597)	1532 (1521–1543)	1500 (1489–1509)	<0.01	1576 (1562–1597)	1539 (1528–1548)	1509 (1499–1516)	<0.01
Stiffness index	103 (95–114)	80 (75–84)	63 (58–66)	<0.01	99 (91–110)	77 (73–81)	61 (57–64)	<0.01
Osteoporosis, %^†^	1.3	2.9	8.2	<0.01	1.9	8.5	21.7	<0.01
IMT, cm	0.61 (0.51–0.73)	0.63 (0.54–0.75)	0.66 (0.56–0.80)	<0.01	0.54 (0.48–0.64)	0.61 (0.51–0.71)	0.68 (0.57–0.78)	<0.01
Plaques, %	43.1	55.2	77.1	<0.01	28.3	44.0	64.6	<0.01
Number of arterial segments with plaque	0 (0–2)	1 (0–2)	2 (1–3)	<0.01	0 (0-1)	0 (0–2)	1 (0–2)	<0.01

BMI: body mass index; BUA: broadband ultrasound attenuation; SOS: speed of sound; IMT: intima-media thickness; 25OHD: 25-hydroxy vitamin D. Data are median (1st–3rd quartiles) or proportions. Group differences were tested with the Kruskal-Wallis or chi-squared tests. ^∗^25OHD: men—1013 missing, women—1127 missing. ^†^Self-reported osteoporosis: men—51 missing, women—63 missing.

**Table 2:** Associations between a decrease in QUS-based parameters and IMT.

Exposure	Adjustment	Men			Women		
		*β*-Coefficient	SE	*p*	*β*-Coefficient	SE	*p*
BUA	Unadjusted	0.009	0.003	<0.01	0.034	0.003	<0.01
SOS		0.020	0.003	<0.01	0.038	0.002	<0.01
Stiffness index		0.016	0.003	<0.01	0.039	0.002	<0.01
Risk: medium versus low		0.022	0.007	<0.01	0.053	0.005	<0.01
Risk: high versus low		0.052	0.148	<0.01	0.113	0.010	<0.01
BUA	Fully adjusted	−0.002	0.002	0.32	−0.003	0.002	0.13
SOS		−0.003	0.002	0.28	−0.000	0.002	0.87
Stiffness index		−0.003	0.002	0.25	−0.002	0.002	0.34
Risk: medium versus low		−0.004	0.004	0.34	−0.003	0.004	0.50
Risk: high versus low		−0.010	0.008	0.19	−0.007	0.008	0.38

BUA: broadband ultrasound attenuation; IMT: intima-media thickness; QUS: quantitative ultrasound; SD: standard deviation; SE: standard error; SOS: speed of sound. *β*-Coefficients, standard errors (SE), and *p* values from linear regression models. For BUA, SOS, and stiffness index, a one standard deviation decrease was modelled. A one standard deviation of BUA for men and women: 13.7 and 14.7 dB/MHz; SOS: 37.5 and 33.5 m/s; stiffness index: 18.1 and 17.5. Full adjustment for age, body mass index, smoking status, physical inactivity, risky alcohol consumption, diabetes mellitus, dyslipidemia, hypertension, and, in women, additionally intake of estrogens (oral contraceptives or hormone replacement therapy) and years since menopause.

**Table 3:** Associations between a decrease in QUS-based parameters and plaques.

Exposure	Adjustment	Odds ratio (95% confidence interval)	
		Men	Women
BUA	Unadjusted	1.32 (1.22–1.42)	1.68 (1.54–1.82)
SOS		1.46 (1.35–1.59)	1.74 (1.59–1.89)
Stiffness index		1.43 (1.33–1.55)	1.80 (1.65–1.96)
Risk: medium versus low		1.62 (1.37–1.93)	1.99 (1.69–2.35)
Risk: high versus low		4.43 (2.87–6.81)	4.61 (3.36–6.32)
BUA	Fully adjusted	1.23 (1.11–1.37)	0.98 (0.88–1.10)
SOS		1.20 (1.08–1.34)	1.01 (0.90–1.12)
Stiffness index		1.24 (1.11–1.38)	1.00 (0.90–1.11)
Risk: medium versus low		1.24 (0.99–1.55)	0.90 (0.73–1.20)
Risk: high versus low		2.93 (1.70–5.06)	0.93 (0.63–1.38)

BUA: broadband ultrasound attenuation; QUS: quantitative ultrasound; SOS: speed of sound. Odds ratios and 95% confidence intervals from logistic regression models. For BUA, SOS, and stiffness index, a one standard deviation decrease was modelled. One standard deviation of BUA for men and women: 13.7 and 14.7 dB/MHz; SOS: 37.5 and 33.5 m/s; stiffness index: 18.1 and 17.5. Full adjustment for age, body mass index, smoking status, physical inactivity, risky alcohol consumption, diabetes mellitus, dyslipidemia, hypertension, and, in women, additionally intake of estrogens (oral contraceptives or hormone replacement therapy) and years since menopause.
