# Supplementary material for: The Association between Bone Quality and Atherosclerosis: Results from Two Large Population-Based Studies
Source: Int J Endocrinol. 2017 Aug 9;2017:3946569. doi: 10.1155/2017/3946569 (PMC5568612; doi:10.1155/2017/3946569)
Supplement: Supplementary file 1 — Supplemental Figure 1. Selection of the study populations. Supplemental Table 1. Characteristics of the ABI study population. Supplemental Table 2. Associations between a decrease in QUS-based parameters and ABI. [file 3946569.f1.pdf]

Supplemental Material

Supplemental Fig 1. Selection of the study populations.

ABI, ankle-brachial-index; BMI, body mass index; eGFR, estimated glomerular filtration rate; IMT, intima media thickness; QUS, quantitative ultrasound; SERM, selective estrogen receptor modulators; SHIP, Study of Health in Pomerania; SI, stiffness index; SOS, speed of sound

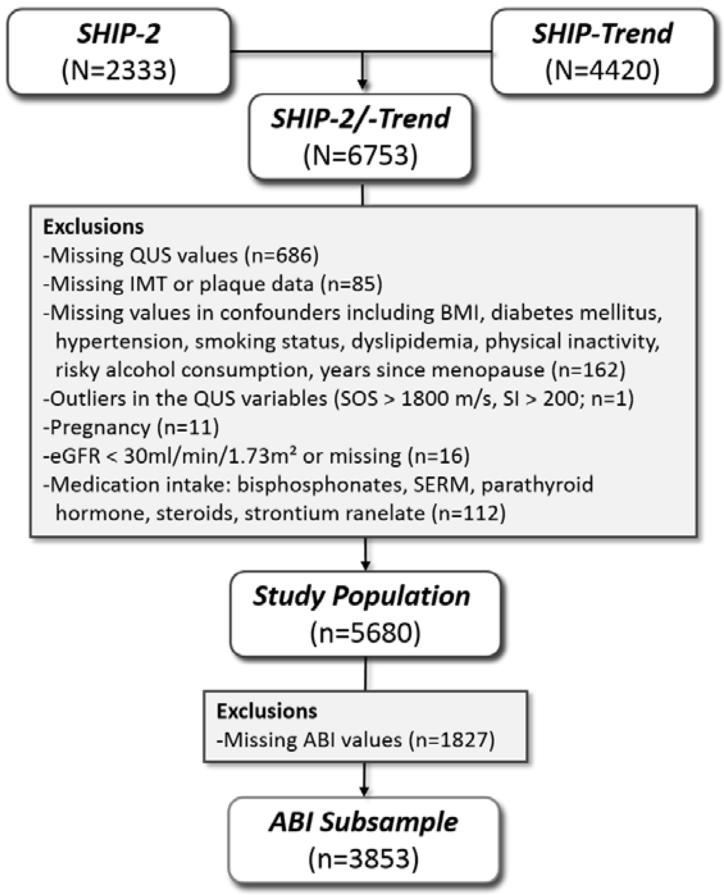

Supplemental Tab 1. Characteristics of the ABI study population

Characteristics	Risk for osteoporotic fractures - Men			Risk for osteoporotic fractures - Women		
	**Low (n=2068)**	**Medium (n=780)**	**High (n=131)**	**Low (n=1991)**	**Medium (n=1029)**	**High (n=191)**
Age, years	52 (41-64)	57 (45-68)	61 (50-70)	48 (39-58)	59 (49-68)	65 (59-73)
BMI, kg/m²	28.2 (25.8-31.1)	28.1 (25.6-30.5)	27.8 (25.1-30.7)	26.6 (23.4-30.8)	26.6 (23.7-30.3)	26.6 (23.6-29.3)
Current Smoker, %	25.3	29.3	42.6	26.3	21.3	17.0
Physically inactive, %	47.1	55.3	67.4	48.4	46.0	43.8
Risky alcohol consumption, %	13.2	13.7	21.8	3.2	2.4	1.0
Diabetes mellitus, %	12.7	16.9	13.4	10.1	13.5	13.6
Dyslipidemia, %	67.4	71.2	70.2	49.2	60.3	66.5
Hypertension, %	72.2	74.8	78.8	45.0	60.4	68.6
BUA, dB/MHz	123 (116-131)	105 (101-109)	94 (90-97)	116 (109-125)	99 (94-103)	86 (83-91)
SOS, m/s	1576 (1560-1598)	1532 (1521-1543)	1500 (1489-1509)	1576 (1561-1596)	1538 (1528-1548)	1509 (1502-1518)
Stiffness Index	103 (95-114)	80 (75-84)	63 (60-66)	98 (91-110)	77 (73-82)	62 (58-64)
Osteoporosis, %*	1.4	2.3	11.4	2.3	8.7	22.5
ABI	1.12 (1.07-1.19)	1.13 (1.07-1.19)	1.12 (1.04-1.21)	1.11 (1.07-1.18)	1.13 (1.08-1.19)	1.13 (1.08-1.18)
ABI ≤0.9, %	2.62	3.35	2.91	0.55	0.37	2.76

ABI, ankle-brachial-index; BMI, body mass index; BUA, broadband-ultrasound-attenuation; SOS, speed of sound
Data are median (1st-3rd quartile) or proportions.
The data were weighted for non-participation in the ABI examination.
*self-reported osteoporosis: men – 60 missings, women 72 missings

Supplemental Tab 2. Associations between a decrease in QUS-based parameters and ABI

Exposure	Adjustment	Men			Women		
		**ß-coefficient**	**SE**	**p**	**ß-coefficient**	**SE**	**p**
BUA	Unadjusted	-0.0015	0.0027	0.58	0.0066	0.0022	<0.01
SOS		-0.0003	0.0027	0.91	0.0068	0.0022	<0.01
Stiffness Index		-0.0009	0.0027	0.73	0.0073	0.0022	<0.01
Risk: Medium vs. Low		0.0020	0.0062	0.75	0.0166	0.0048	<0.01
Risk: High vs. Low		-0.0061	0.0133	0.65	0.0019	0.0095	0.84
BUA	Fully adjusted	0.0015	0.0027	0.57	0.0026	0.0024	0.28
SOS		0.0015	0.0027	0.59	0.0040	0.0024	0.10
Stiffness Index		0.0017	0.0028	0.54	0.0038	0.0025	0.13
Risk: Medium vs. Low		0.0050	0.0062	0.42	0.0099	0.0051	0.05
Risk: High vs. Low		0.0021	0.0133	0.87	-0.0097	0.0100	0.33

ABI; ankle-brachial-index; BUA, broadband-ultrasound-attenuation; QUS, quantitative ultrasound; SE, standard error; SOS, speed of sound

ß-coefficients, standard errors (SE) and p-values from linear regression models. For BUA, SOS and Stiffness Index a one standard deviation decrease was modelled. One standard deviation of BUA for men and women: 13.6 and 14.7 dB/MHz; SOS: 37.4 and 33.5 m/S; stiffness index: 18.0 and 17.6

Full adjustment for age, body mass index, smoking status, physical inactivity, risky alcohol consumption, diabetes mellitus, dyslipidemia, hypertension and in women additionally for intake of estrogens (oral contraceptives or hormone replacement therapy) and years since menopause.

The data were weighted for nonparticipation in the ABI examination.
